# Coexisting Charge Density Waves in Twisted Bilayer NbSe_2_

**DOI:** 10.1021/acs.nanolett.4c02750

**Published:** 2024-09-19

**Authors:** Christopher T. S. Cheung, Zachary A. H. Goodwin, Yixuan Han, Jiong Lu, Arash A. Mostofi, Johannes Lischner

**Affiliations:** † Departments of Physics and Materials and the Thomas Young Center for Theory and Simulation of Materials, Imperial College London, South Kensington Campus, London SW7 2AZ, U.K.; ‡ John A. Paulson School of Engineering and Applied Sciences, Harvard University, Cambridge, Massachusetts 02138, United States; § Institute for Functional Intelligent Materials, National University of Singapore, Singapore 117544, Singapore

**Keywords:** charge density waves, twistronics, first-principles simulation, 2D materials

## Abstract

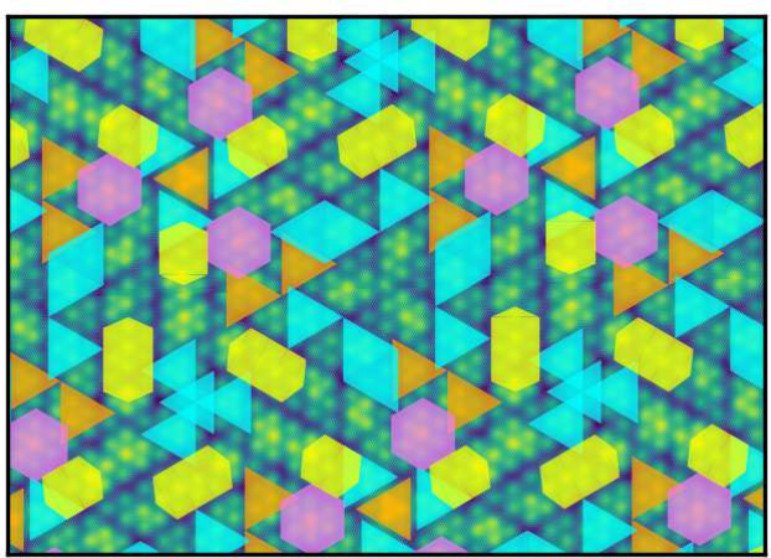

Twisted bilayers of 2D materials have emerged as a tunable platform for studying broken symmetry phases. While most interest has been focused toward emergent states in systems whose constituent monolayers do not feature broken symmetry states, assembling monolayers that exhibit ordered states into twisted bilayers can also give rise to interesting phenomena. Here, we use first-principles density-functional theory calculations to study the atomic structure of twisted bilayer NbSe_2_ whose constituent monolayers feature a charge density wave. We find that different charge density wave states coexist in the ground state of the twisted bilayer: monolayer-like 3 × 3 triangular and hexagonal charge density waves are observed in low-energy stacking regions, while stripe charge density waves are found in the domain walls surrounding the low-energy stacking regions. These predictions, which can be tested by scanning tunneling microscopy experiments, highlight the potential to create complex charge density wave ground states in twisted bilayer systems.

Twisted bilayers of two-dimensional (2D) materials have been intensely studied in recent years because of their novel electronic,^1−12^ optical,^13−17^ and vibrational properties.^18,19^ These materials are formed by vertically stacking two monolayers and then rotating them relative to each other. This gives rise to an emergent moiré pattern whose lattice constant depends on the twist angle.^20,21^

To date, many studies on twisted bilayer materials have focused on their emergent broken-symmetry states, i.e., states that are absent in the monolayer. For example, magic-angle twisted bilayer graphene exhibits correlated insulator states and superconductivity, which are not found in the constituent graphene sheets.^2,3^ Recently, there has been increasing interest in twisted bilayer materials composed of monolayers with broken-symmetry states, such as bilayers of magnetic CrI_3_.^22−26^

Another class of broken-symmetry states that can be found in monolayers is charge-density waves (CDWs). For example, monolayer NbSe_2_ adopts a 3 × 3 triangular CDW in which some Nb atoms move toward an interstitial site.^27^ However, other types of CDWs have also been predicted and observed, such as 2 × 2, 2 × 3, hexagonal and chalcogen-centered triangular 3 × 3 CDWs,^28,29^ and stripe-like 4 × 4 or  ×  CDWs.^30−32^ The various CDWs can be viewed as different linear combinations of the three soft phonon modes of the high-symmetry phase.^33−38^

Importantly, the condensation energies of the various CDWs in the monolayer differ only by a few meV, and their relative stability is highly sensitive to external perturbations, such as strain and doping.^29,32,39^ It is therefore interesting to ask what happens to the CDW when a twisted bilayer is formed.

Based on earlier models of CDWs in the monolayer,^33−38^ Goodwin and Fal’ko developed a Landau free energy approach based on a number of simplifying assumptions to understand CDWs in a twisted bilayer system at small twist angles.^40^ In particular, they assumed that CDW is not affected by strain. They predicted that certain CDWs (such as √3 × √3) can propagate through the moiré system without geometric constraints, while other CDW (such as 3 × 3 or 2 × 2) only exist in specific regions of the moiré unit cell. This is in agreement with the experimental observation of a 2 × 2 CDW in some regions of a twisted TiTe_2_ monolayer on a TiSe_2_ bilayer, while other regions remain in the normal state.^41^ Later, McHugh et al. developed a multiscale model based on first-principles DFT calculations of untwisted bilayers to study the relaxed structure of marginally twisted bilayer NbSe_2_, but they also did not include the effect of strain on the CDW.^42^

In this paper, we present results for the relaxed atomic structure of antiparallel twisted bilayer NbSe_2_ for a twist angle of 3.14° (with a moiré unit cell containing 1986 atoms) using *ab initio* density-functional theory,^43^ which fully captures the coupling between CDWs and strain. We find that the twisted bilayer NbSe_2_ exhibits significant in-plane and out-of-plane atomic relaxations. We also observe that different CDW motifs coexist in different regions of the moiré unit cell: low-energy MM regions are dominated by filled-center, hollow-center, and hexagonal 3 × 3 CDWs, while the surrounding domain wall regions exhibit a stripe CDW whose formation is a consequence of local strain which suppresses contributions to the CDW from all but a single wavevector.

We start by discussing the atomic structure of the relaxed twisted NbSe_2_ bilayer and compare our findings to twisted bilayer MoSe_2_, which does not feature a CDW in its monolayer form and serves as a useful reference point for understanding the unconventional behavior of NbSe_2_.

Figures 1(a) and (b) show the interlayer separation (ILS) between the metal atom layers with the high-symmetry stacking regions indicated by red symbols. In agreement with many previous studies,^44−49^ we find that the relaxed twisted bilayers exhibit a corrugated structure.

**Figure 1:**
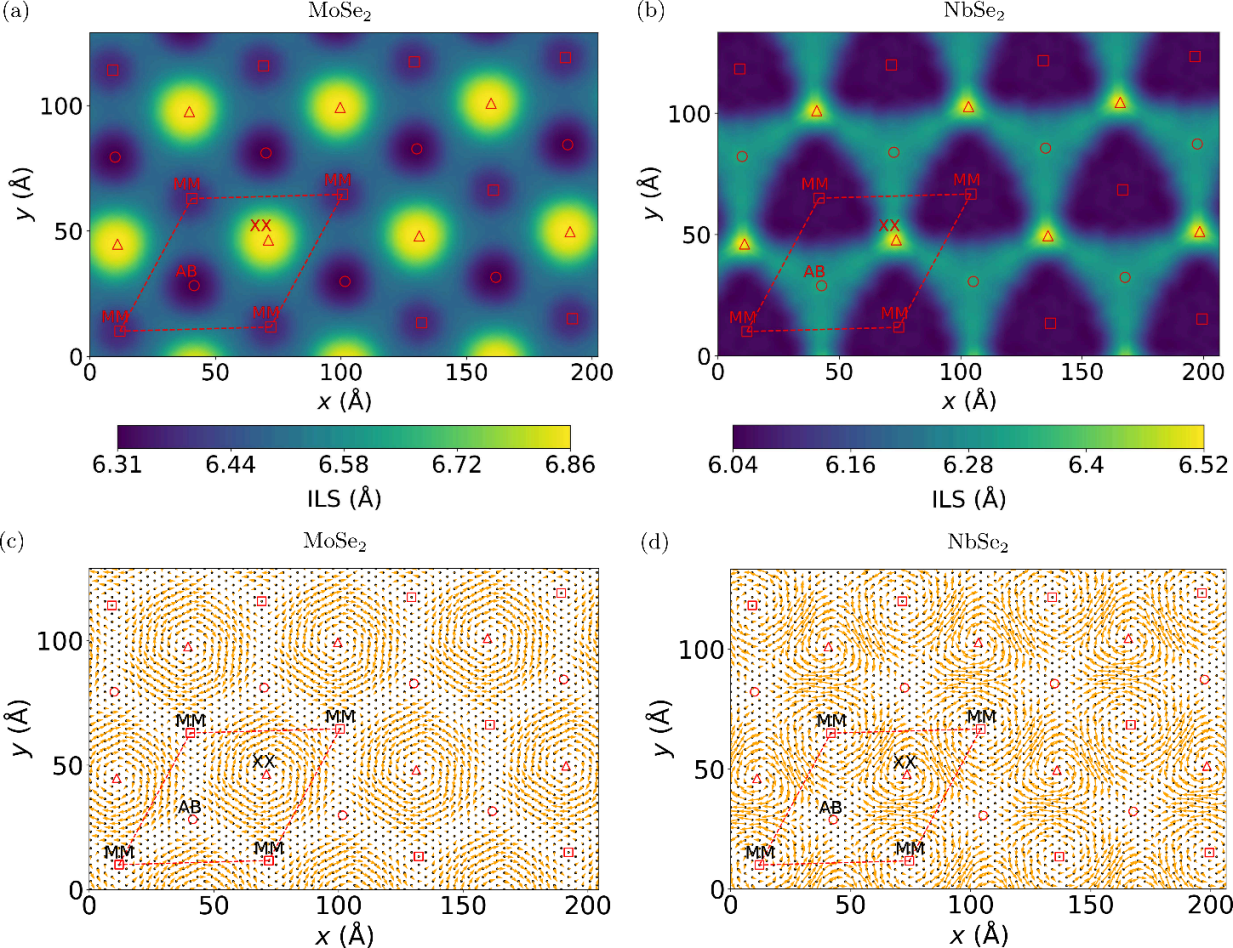
In-plane (bottom panels) and out-of-plane (top panels) atomic relaxations of twisted bilayers MoSe_2_ (left panels) and NbSe_2_ (right panels). The interlayer separation (ILS) between the metal atom layers in (a) twisted bilayer MoSe_2_ and (b) twisted bilayer NbSe_2_ at a twist angle of 3.14°. In-plane displacements of metal atoms relative to the unrelaxed initial structure in the bottom layer for (c) twisted bilayer MoSe_2_ and (d) twisted bilayer NbSe_2_. The red circles denote the centers of the AB stacking regions (metal on top of chalcogen and *viceversa*), the red squares denote the centers of the MM stacking regions (metal on top of metal), and the red triangles denote the centers of the XX stacking regions (chalcogen on top of chalcogen). The red parallelogram marks the repeating unit used in the DFT calculation.

For twisted bilayer MoSe_2_ (shown in Figure 1(a)), the ILS reaches a maximum of 6.86 Å in the XX stacking region (in which the chalcogen atoms of the top layer are directly above the chalcogen atoms of the bottom layer) and a minimum of 6.31 Å at the AB stacking region (in which the metal atoms of one layer have the same in-plane positions as the chalcogen atoms of the other layer). The ILS in the MM stacking region (in which the metal atoms of the top layer are directly above the metal atoms of the bottom layer) is 6.37 Å, which is only slightly larger than the ILS of the AB stacking region. We note that the interlayer separations at the AB, MM, and XX points are close to those found in the corresponding untwisted bilayers.^44^

In twisted bilayer NbSe_2_ (shown in Figure 1(b)), the ILS is also largest at the XX stacking region, with a value of 6.57 Å. However, the ILS of the AB point (6.31 Å) is significantly larger than that of the MM point (6.03 Å). Again, the interlayer separations at different stacking points are similar to those of the untwisted bilayers (6.57 Å for XX stacking, 6.25 Å for AB stacking, and 6.05 Å for MM stacking).

The different relative ordering of the interlayer separation of the MM stacking regions and the AB stacking regions in NbSe_2_ compared to MoSe_2_ results in a qualitatively different interlayer separation landscape in the two twisted bilayer materials, as seen in Figures 1(a) and (b). In twisted bilayer NbSe_2_, a triangular plateau of relatively constant interlayer separation emerges, which is centered on the AB point and has corners at the XX points. Another triangular region of the small interlayer separation is centered on the MM points. Its size is significantly larger than the MM centered spherical region of small ILS in twisted MoSe_2_. Our findings are consistent with the results of McHugh et al., who used a continuum elasticity approach.^42^

Figures 1(c) and (d) compare the in-plane atomic displacements (relative to the unrelaxed starting structure) of the bottom layers of twisted bilayer NbSe_2_ and twisted bilayer MoSe_2_ (the results for the top layers are shown in Supplementary Figure S1). In twisted bilayer MoSe_2_, large vortex-like displacement patterns are observed, which are centered at the points of XX stacking. In between these vortices are domain walls connecting the MM and AB stacking points where the displacements are small. These findings can be understood by considering the energetics of the different stackings: since XX stacking is energetically unfavorable, atoms move to minimize the area of XX stacking regions, resulting in the formation of vortices. This, in turn, leads to shear strain solitons around the vortex, giving rise to domain walls.^44,45^

In twisted bilayer NbSe_2_, we also find vortices centered on points of XX stacking. However, their shape is less circular than in twisted MoSe_2_ and the magnitude of the displacements along a circular path centered at the XX point is strongly modulated. In twisted bilayer MoSe_2_, the magnitudes of the in-plane displacements along the vortices are up to 0.17 Å and relatively uniform along circular paths centered on the XX points. In contrast, in twisted bilayer NbSe_2_, the magnitudes of the displacements are both larger and exhibit more variation; for example, they can be as large as 0.44 Å where the circular displacement pattern intersects the lines connecting the vortex center to the MM points and 0.25 Å on the lines connecting the vortex center to the AB points.

The different behavior of the in-plane displacements of twisted bilayer NbSe_2_ and MoSe_2_ is a consequence of their different out-of-plane relaxations. While the interlayer separation of twisted bilayer MoSe_2_ is approximately constant along a circle centered at the XX point, this is not the case for twisted bilayer NbSe_2_, as can be seen in Figures 1(a) and (b). In particular, in NbSe_2_ the ILS is small along lines joining XX and MM points, which gives rise to a strong steric repulsion, which in turn drives large in-plane displacements. In contrast, the ILS is larger along lines joining XX and AB points, giving rise to weaker steric repulsion and smaller in-plane displacements.

In contrast to the monolayer, it is difficult in a twisted bilayer to ascertain the presence of a CDW from the atomic displacement vectors relative to the initial structure without a CDW: In a twisted bilayer system, large in-plane atomic relaxations occur to reduce the area of high-energy stacking regions and increase the area of low-energy stacking regions. These displacements are often much larger than the displacements that give rise to the formation of CDWs, and this makes the identification of a CDW challenging.

As an alternative way to visualize the presence of CDWs in twisted bilayer NbSe_2_, we use the smeared atomic density of Nb atoms obtained by associating a Gaussian function with each Nb site; see Supporting Information for details. For the monolayer, the formation of a 3 × 3 triangular CDW can easily be ascertained from an inspection of the smeared Nb density; see Figure 2(e). The 3 × 3 triangular CDW is characterized by two sets of triangles: larger six-atom triangles and smaller three-atom triangles. If the triangles (highlighted in cyan color) are centered on interstitial sites, the CDW is referred to as a hollow-center 3 × 3 CDW; see Figure 2(a). In contrast, if the triangles (highlighted in orange color) are centered on the Se atoms, it is called a filled-center 3 × 3 CDW;^27^ see Figure 2(b). The bright triangles in the smeared atomic density are separated by dark blue lines running along three directions with angles of 120° between them. These three directions correspond to the three wave vectors **q**_*n*_ (with *n* = 1, 2, 3) at which the phonon spectrum of the unrelaxed monolayer exhibits instabilities;^27,50^ see discussion below. Another CDW that has been predicted to be metastable in the monolayer^29^ is the hexagonal CDW, where the nearest six nearest-neighbor Nb atoms move toward the central Nb atom, as shown in Figure 2(c), giving rise to hexagons in the smeared atomic density (highlighted by purple color). Finally, one-dimensional stripe CDWs (highlighted in yellow color; see Figure 2(d)) have been predicted to occur in the monolayer under applied strain.^28,31^

**Figure 2:**
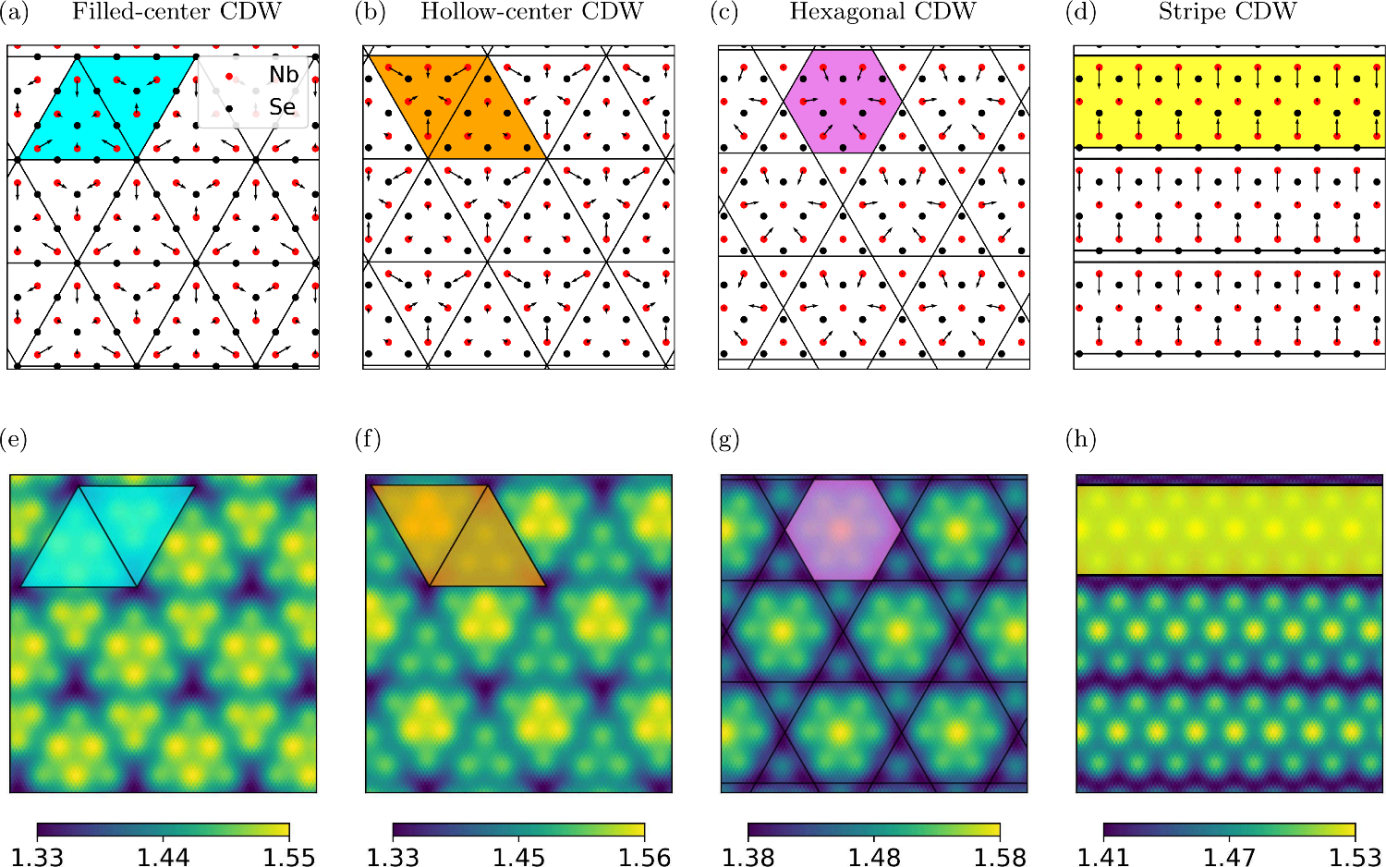
Atomic displacements and smeared Nb atomic densities of different charge density wave states. Atomic displacements of Nb atoms (red circles) due to a (a) filled-center CDW, (b) hollow-center CDW, (c) hexagonal CDW, and (d) stripe CDW in monolayer NbSe_2_. The Se atoms (black circle) are also shown. Smeared Nb atomic densities of (e) filled-center CDW, (f) hollow-center CDW, (g) hexagonal CDW, and (h) stripe CDW in monolayer NbSe_2_. The colored motifs used for highlighting the CDW motifs in Figures 3(b) and (d) are demonstrated in these figures. We use (a and e) cyan triangles for three- or six-atom filled-center CDW, (b and f) orange triangles for three- or six-atom hollow-center CDW, (c and g) purple hexagon for hexagonal CDW, and (d and h) yellow strips for stripe CDW.

Figures 3(a) and (c) show the smeared Nb atomic densities of the bottom and top layers of twisted bilayer NbSe_2_, respectively. In contrast to the monolayer, which exhibits a uniform triangular 3 × 3 CDW phase in its ground state, a variety of different CDW-like motifs can be observed in the twisted bilayer. To characterize these motifs, we have locally projected the displacements patterns obtained from our DFT relaxations onto reference displacement patterns characterizing the various CDWs shown in Figure 2; see Supporting Information for details. In the MM stacking region (which has the lowest energy and therefore increases in size upon relaxation), we find that hollow-center (orange triangles), filled-center (cyan triangles), and hexagonal (purple hexagons) CDWs coexist, as can be seen in Figures 3(b) and (d). In the bottom layer (Figure 3(b)), the MM center is surrounded by filled-center CDWs that are interspersed by a few hollow-center and hexagonal CDW motifs. At the center of the AB region, a hollow-center CDW motif is found that is surrounded by three filled-center CDW motifs, and a filled-center center motif is found as the center of the XX region. In the top layer (Figure 3(d)), a hexagonal CDW motif is found at the MM center, which is surrounded by hollow-center CDWs and a few filled-center CDW motifs. As in the bottom layer, a hollow-center (filled-center) CDW motif is located at the AB (XX) center. For comparison, Supplementary Figure S2 shows the smeared Mo atomic density of relaxed twisted bilayer MoSe_2_. In this system, no CDW motifs can be observed.

**Figure 3:**
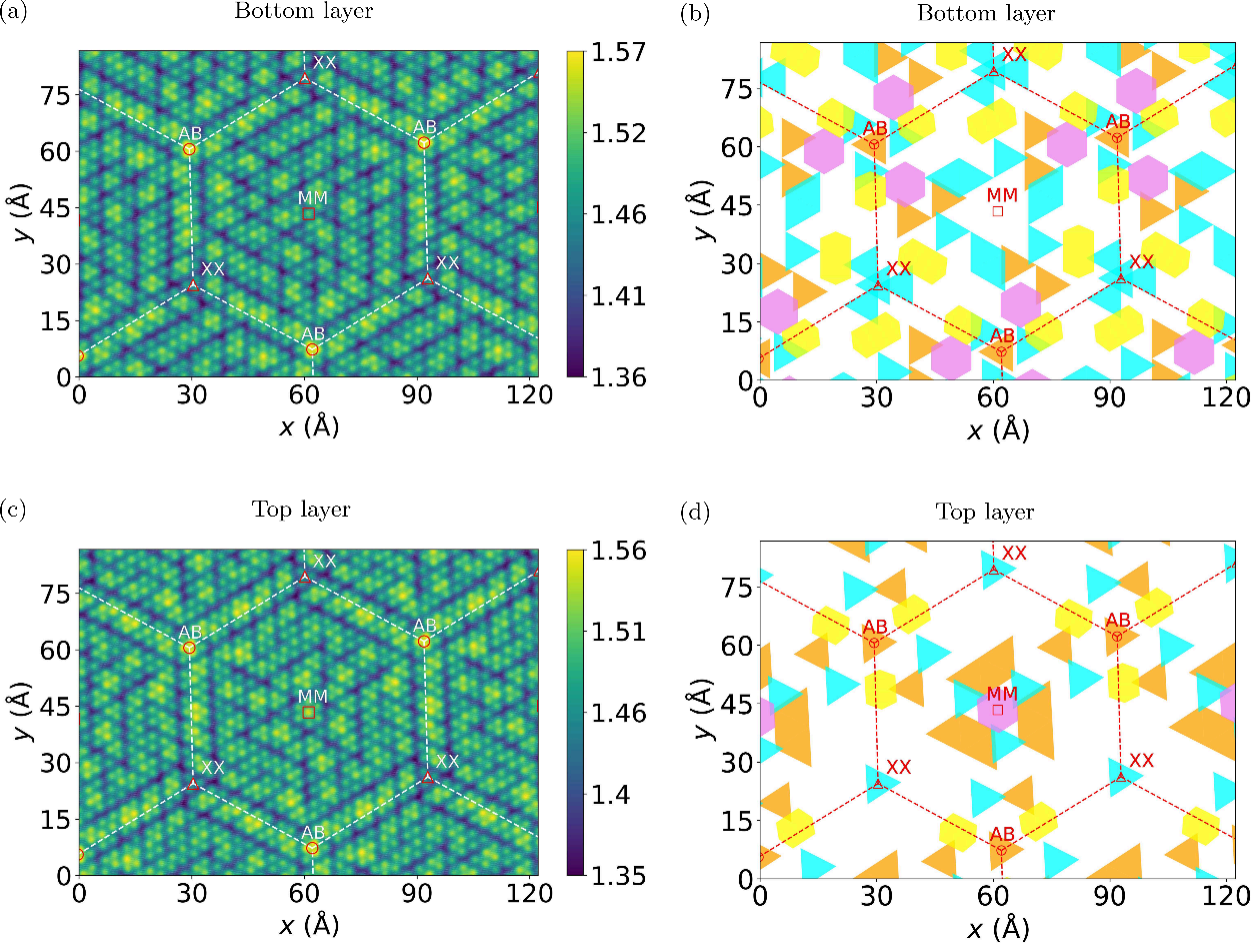
Coexisting charge density waves in the twisted bilayer NbSe_2_. Smeared Nb atomic density of (a) the bottom layer and (c) the top layer of twisted bilayer NbSe_2_ at a twist angle of 3.14°. The hollow-center CDWs (orange triangles), filled-center CDWs (cyan triangles), hexagonal CDWs (violet hexagons), and stripe CDWs (yellow symbols) are indicated in (b) for the bottom layer and in (d) for the top layer. The red circles denote the centers of the AB stacking regions (metal on top of chalcogen and *viceversa*); the red squares denote the centers of the MM stacking region (metal on top of metal); and the red triangles denote the centers of the XX stacking regions (chalcogen on top of chalcogen). The white dashed lines join the AB and XX centers for visual aid in parts (a) and (c). In parts (b) and (d), red dashed lines are used.

In Figure 4, we superimpose the CDW motifs of the top and bottom layers in and around the MM region. We observe that a hollow-center CDW motif (orange triangle) is found in the top layer whenever a filled-center CDW motif (cyan triangle) is located directly underneath the bottom layer and *vice versa*. This means that Nb atoms in the two layers move in opposite directions; that is, if a Nb atom in the bottom layer moves toward an interstitial site, its nearest neighbor in the top layer moves toward a Se site and *vice versa*. This coordinated motion of Nb atoms in both layers reduces the steric repulsion between Se atoms: the in-plane movement of the Nb atoms forming CDWs induces a corresponding out-of-plane displacement of the neighboring Se atoms. If Nb atoms in both layers move in the same direction, the neighboring Se atoms would move toward each other, increasing the total energy due to steric repulsion.

**Figure 4:**
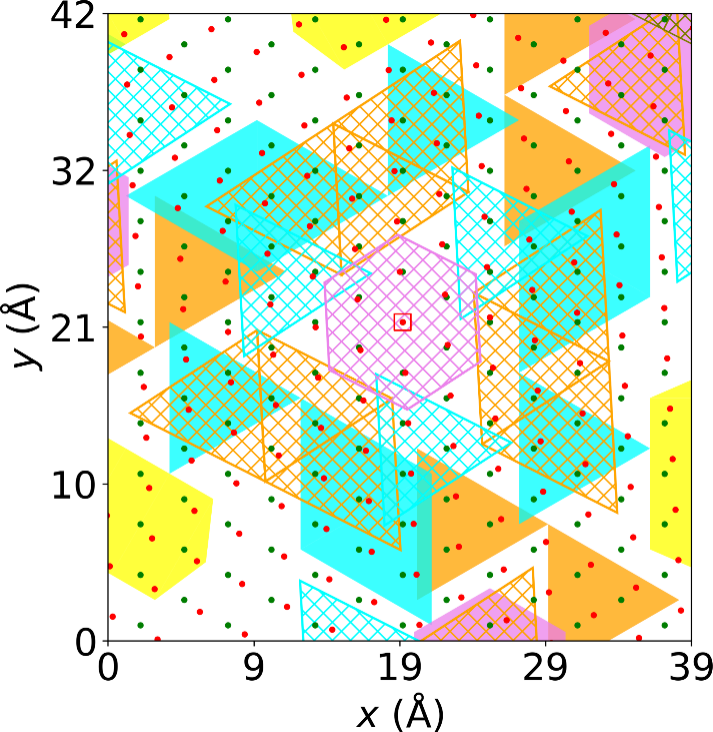
Interlayer stacking of CDW motifs in twisted bilayer NbSe_2_ in the MM region. The green circles represent the Nb atoms in the bottom layer, and the red circles represent the Nb atoms in the top layer. The cyan (orange) solid triangles represent the filled-center (hollow-center) CDW motifs in the bottom layer. Purple solid hexagons represent hexagonal CDW motifs in the bottom layer. The cyan (orange) hatched triangles and purple hatched hexagons represent the filled-center (hollow-center) and hexagonal CDW motifs in the top layer. The yellow solid strips show the stripe CDW in the bottom layer.

Surrounding the regions of MM stacking are domain walls that connect the XX points to the AB points. In these domain wall regions, we observe stripe CDWs (yellow strips), which exhibit an extended character along the direction of the domain wall. The formation of 1D CDWs along the domain walls is a consequence of the local strain. In particular, we find that if the atoms experience a local strain along one of the three CDW wavevectors {**q**_*n*_}, the contribution to the CDW from this wavevector is suppressed. Figure 5 shows the strain along each of the three CDW wavevectors (see Supporting Information for details). This demonstrates that a one-dimensional CDW with a specific wavevector, for example, **q**_1_, occurs in regions where there is no strain along **q**_1_, but significant strain along **q**_2_ and **q**_3_. This is consistent with previous findings that nonuniform strain induces one-dimensional stripe charge density waves.^32,39^

**Figure 5:**
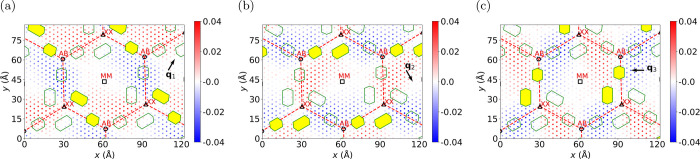
Local strain and stripe charge density waves in twisted bilayer NbSe_2_. Distribution of strain in the bottom layer along each of the three charge density wave vectors: (a) **q**_1_, (b) **q**_2_, and (c) **q**_3_. Green strips indicate the stripe CDWs. In each panel, the hexagons describing stripe CDWs characterized by the wave vector **q**_1_ (a), **q**_2_ (b), and **q**_3_ (c) are colored in yellow. The solid circles represent the magnitude of the strain. The black circles denote the centers of the AB stacking regions (metal on top of chalcogen and *vice versa*); the black squares denote the centers of the MM stacking regions (metal on top of metal); and the black triangles denote the centers of the XX stacking regions (chalcogen on top of chalcogen). The red dashed lines join the AB and XX centers for visual aid.

Our calculations suggest the following picture for the fate of the CDW in twisted bilayer NbSe_2_: atomic relaxations driven by the energetics of different stacking arrangements give rise to a strain pattern that acts as a template for the formation of CDWs. Since the strain pattern is commensurate with the moiré unit cell, the resulting CDW pattern will also have this property (even if the periodicity of the moiré unit cell is not commensurate with 3 × 3 CDW, which has the lowest energy in the monolayer). To further support this conclusion, we have carried out a calculation for a 3 × 3 supercell (which is commensurate with the 3 × 3 CDW) of a twisted bilayer NbSe_2_ at a twist angle of θ = 9.43°. As expected, we find that each of the nine moiré unit cells contained in the supercell have the same atomic structure; see Supplementary Figure S3. These findings highlights the importance of strain on the CDW in a twisted bilayer. We note that a previous study by Goodwin and Falko neglected this effect.^40^

Based on our findings, we expect the following scenario for smaller twist angles (which are difficult to access with first-principles methods): the low-energy MM-stacking regions grow in size and feature 3 × 3 triangular CDWs as the twist angle is reduced. The strain becomes strongly localized at the boundaries of MM-stacked regions and favors the formation of stripe CDWs.

The predictions from our calculations can be tested with STM experiments. To aid the comparison with such experiments, we have included simulated STM images in the Supporting Information.

In this study, we investigated the relaxed atomic structure of twisted bilayer NbSe_2_ using large-scale *ab initio* density-functional theory calculations. We observe an interesting interplay of in-plane and out-of-plane relaxations as well as the formation of coexisting charge density waves. In particular, low-energy stacking regions exhibit both triangular and hexagonal charge density waves with a 3 × 3 periodicity similar to the monolayer. Steric repulsion between Se atoms results in the stacking of hollow-center triangular CDWs on top of filled-center triangular CDWs and *viceversa*. In the domain walls surrounding the low-energy stacking domains stripe CDWs are formed as a consequence of anisotropic localized strain, which suppresses contributions to the CDW from all but one wavevector. Our work sheds light on the complex interplay of atomic displacements driven by stacking energetics and those driven by the formation of charge density waves and also challenges the fundamental assumptions of previous investigations in which the interaction of strain with CDWs was neglected. Our predictions can be tested by scanning tunneling microscopy experiments. Besides CDWs, NbSe_2_ also exhibits superconductivity at low temperatures. Both the transition temperature and the symmetry of the superconducting state depend on the number of layers and are influenced by the properties of the CDW state.^27,51−53^ Therefore, the insights from our study provide a starting point for future investigations of superconductivity in twisted bilayers with CDW order.

## Supporting Information Available

Additional details on the methodology, computational details, additional relaxation results, and simulated STM images (PDF)
