# Supplementary material for: Coexisting Charge Density Waves in Twisted Bilayer NbSe2
Source: Nano Lett. 2024 Sep 19;24(39):12088–94. doi: 10.1021/acs.nanolett.4c02750 (PMC11450987; doi:10.1021/acs.nanolett.4c02750)
Supplement: Supplementary file 1 — nl4c02750_si_001.pdf [file nl4c02750_si_001.pdf]

Supplementary Information: Coexisting charge density waves in twisted bilayer $NbSe_2$

Christopher T. S. Cheung,[1] Zachary A. H. Goodwin,[2] Yixuan Han,[3] Jiong Lu,[3] Arash A. Mostofi,[1] and Johannes Lischner[1]

[1] *Departments of Physics and Materials and the Thomas Young center for Theory and Simulation of Materials, Imperial College London, South Kensington Campus, London SW7 2AZ, UK*

[2] *John A. Paulson School of Engineering and Applied Sciences, Harvard University, Cambridge, MA 02138, USA*

[3] *Institute for Functional Intelligent Materials, National University of Singapore, Singapore 117544, Singapore*

METHODS

Construction of the moiré unit cell

To construct the starting configuration for the atomic relaxations, we stack and twist two flat anti-parallel $NbSe_2$ monolayers that do not feature a charge density wave. The resulting moiré unit cell is spanned by the lattice vectors $\mathbf{t}_1$ and $\mathbf{t}_2$ given by

$$\begin{aligned}\mathbf{t}_1 &= n\mathbf{a}_1 + m\mathbf{a}_2,\\ \mathbf{t}_2 &= -m\mathbf{a}_1 + (n+m)\mathbf{a}_2,\end{aligned} \tag{1}$$

where $\mathbf{a}_1 = a_0/2(\sqrt{3}, 1, 0)$ and $\mathbf{a}_2 = a_0/2(\sqrt{3}, -1, 0)$, and a_0 is the lattice constant of monolayer $NbSe_2$ without any charge density wave. The value of the lattice constant is found by relaxing the $NbSe_2$ unit cell using DFT as described in the next section. We found the equilibrium lattice constant to be 3.44 Å, in good agreement with other values reported in literature [1–3]. The integers n, m are the number of unit cells along each moiré lattice vector. The twist angle is given by $\cos\theta = (n^2 + 4nm + m^2)/[2(n^2 + nm + m^2)]$ [4]. Thus, choosing the pair of integers (n, m) as $(10, 11)$ gives the twist angle of 3.14°. The number of atoms in the moir unit cell is $N = 6(n^2 + nm + m^2)$, which is 1,986 at the chosen twist angle.

Computational parameters

To determine the relaxed atomic structure of twisted bilayer $NbSe_2$, we use ab initio density-functional theory as implemented in the SIESTA code [5]. To capture van der Waals interactions, the exchange-correlation functional developed by Cooper is used [6]. We use Troullier-Martins pseudopotentials [7], a double-ζ polarized basis and Γ-point sampling of the first Brillouin zone. In the self-consistent cycle, the Hamiltonian and the density matrix are converged to 1 μeV and 1×10^{-7} per atom, respectively. The force tolerance is set to 5×10^{-2} eV/Å.

Smeared Nb atomic density

The smeared Nb atomic density for a given layer is the sum of Gaussian densities associated with each Nb atomic site, and is given by

$$\rho(\mathbf{r}) = \sum_{i=1}^{N_{\mathrm{Nb}}} e^{-|\mathbf{r}-\mathbf{R}_i|^2/(2\sigma^2)}, \tag{2}$$

where N_{Nb} is the number of Nb atoms in the layer, $\mathbf{R}_i$ is the relaxed atomic position of the i-th Nb atom, and σ is a parameter that controls the width of the Gaussian. Supplementary Fig. S5 shows the smeared Nb density for different values of σ for a $NbSe_2$ monolayer. For very small values of σ such as 0.2 a_0 (see Supplementary Fig. S5(a)), it is difficult to see whether a CDW is present or not. However, if σ is increased to 0.45 a_0 (see Supplementary Fig. S5(b)), the 3×3 modulation becomes evident. If σ is further increased to 0.9 a_0 (see Supplementary Fig. S5(c)), the atomic resolution is lost, but the 3×3 modulation is still visible. We use $\sigma = 0.45a_0$ in this paper as this choice both provides atomic resolution and enables easy identification of charge density waves.

Order parameter for charge density wave motifs

As described in the main text, the identification of CDW motifs based on the Nb displacement vectors relative to the initial structure is challenging in a twisted bilayer $NbSe_2$. In contrast to the monolayer where the Nb displacement vectors relative to the high-symmetry structure without CDW can be used to assess the presence of a CDW, the displacement vectors in twisted bilayer are dominated by the movement of the atoms to avoid high-energy stacking arrangements.

To determine the smaller local displacements of the Nb atoms due the formation of a CDW, we note that in the high-symmetry phase without CDW the position of a Nb atom coincides with the centroid of its six neighboring Nb

atoms, i.e. the position of a Nb atom is equal to the average of the positions of its nearest Nb neighbors. When a CDW is formed, the Nb atom position no longer coincides with the centroid of the nearest Nb neighbors.

To assess the presence of a CDW in a twisted bilayer, we therefore define the local displacement $\mathbf{u}_i$ of the i-th Nb atom according

$$\mathbf{u}_i = \mathbf{R}_i - \frac{1}{6}\sum_{l=1}^{6}\mathbf{R}_l, \tag{3}$$

where $\mathbf{R}_i$ is the relaxed position of the i-th Nb atom and $\mathbf{R}_l$ are the relaxed positions of its six nearest Nb neighbors. The second term on the right hand side is the centroid of the nearest Nb neighbors. The local displacements of the Nb atoms are shown in Supplementary Figure S6.

To assess the presence of a specific CDW motif (labeled by the index α) in the twisted bilayer, we search through all possible motif centers (labeled by the index i) and project the local displacements of the neighboring Nb atoms (labeled by the index l) onto a reference displacement pattern $\mathbf{u}_l^{\mathrm{ref}(\alpha)}$, shown in Fig. 2(a)-(d). For the filled-center CDWs, the motif centers are the Se atoms. For the hollow-center CDWs, the motif centers are the interstitial sites. For the hexagonal and stripe CDWs, the motif centers are the Nb atoms. The local order parameter for i-th motif center is then obtained as

$$p_i^\alpha = \frac{1}{N_{\mathrm{at}}}\sum_{l=1}^{N_{\mathrm{at}}} p_{il}^\alpha, \tag{4}$$

where N_{at} denotes the number of Nb atoms near the motif center whose displacements are taken into account, and p_{il}^α is the contribution to the total order parameter from l-th Nb atom given by

$$p_{il}^\alpha = \exp\left(-\frac{\arccos\left(\hat{\boldsymbol{u}}_l^{\mathrm{ref}(\alpha)}\cdot\hat{\boldsymbol{u}}_l\right)^2}{2\gamma^2}\right), \tag{5}$$

where γ is an smearing parameter. Here, we use a value of $\pi/8$ for this parameter. Note that $1 \leq p_i^\alpha \leq 0$. We have verified that p_i^α is close to unity if the order parameter is calculated for a monolayer with a uniform CDW.

In a twisted bilayer, we consider a CDW motif of type α to be present at the i-th motif center, if p_i^α exceeds a critical value p_{c}^α.

For the filled-center (hollow-center) CDW motifs, for each Se atom (interstitial site), separate order parameters are calculated for the smaller three-atom triangles ($N_{\mathrm{at}} = 3$) and the larger six-atom triangles ($N_{\mathrm{at}} = 6$). If p_i^α for the three-atom triangle exceeds $p_{\mathrm{c}}^\alpha = 2/3$ (i.e. more than two of the three atoms in the triangle follow the reference displacement pattern) and p_i^α for the six-atom triangle is smaller than $p_{\mathrm{c}}^\alpha = 4/6$ (i.e. fewer than four of the six atoms in the triangle follow the reference displacement pattern), the three-atom motif is assumed to be present. If p_i^α for both the three- and the six-atom triangle exceed $p_{\mathrm{c}}^\alpha = 2/3$, a six-atom triangle is assumed to be present.

For hexagonal CDW motifs, the order parameter is calculated for each Nb atom so the contributions from all neighboring Nb atoms are taken into account ($N_{\mathrm{at}} = 6$). We use $p_{\mathrm{c}}^\alpha = 1/2$ (i.e. more than three of the six Nb atoms follow the reference displacement pattern).

For stripe CDW motifs along a wave vector $\boldsymbol{q}_n$, we consider pairs of Nb atoms ($N_{\mathrm{at}} = 2$) whose distance vector is parallel to $\boldsymbol{q}_n$. If at least three neighboring pairs of Nb atoms have an order parameter exceeding $p_{\mathrm{c}}^\alpha = 1/2$ (i.e. more than one out of the two atoms follow the reference displacement pattern), we consider a stripe CDW to be present.

We have verified that the above procedure reliably identifies the correct CDW motifs. In particular, we have applied this approach to monolayers with different types of CDWs and found that it (i) correctly identifies the CDW type that is present in the system and (ii) does not spuriously identify CDW types which are not present in the system, see Supplementary Fig. S7.

Determining the strain

To estimate the local strain at the i-th Nb atom due to moir relaxations, we partition the total displacement of the Nb atoms from their high-symmetry positions to their relaxed positions into contributions from moir displacements and local displacements. The moir displacements are then given by

$$\mathbf{u}_i^{\mathrm{moir}} = \Delta\mathbf{R}_i - \mathbf{u}_i, \tag{6}$$

with $\Delta\mathbf{R}_i$ denoting the total displacement from the high-symmetry position. The moiré displacements are shown in Supplementary Figure S7.

The local strain along the direction $\hat{\mathbf{q}}_n$ at the i-th Nb atom to moir displacement is then estimated as

$$\boldsymbol{\varepsilon}_i^{(n)} = \left(\frac{\mathbf{u}_{i-1,n}^{\mathrm{moir}} + \mathbf{u}_{i+1,n}^{\mathrm{moir}}}{\sqrt{3}a_0} \right) \cdot \hat{\boldsymbol{q}}_n, \tag{7}$$

where $\sqrt{3}a_0$ denotes the distance between the two Nb atoms in the initial configuration and $\mathbf{u}_{i\pm1,n}^{\mathrm{moir}}$ denote the moiré displacements of the two Nb nearest neighbors of atom i along the direction of $\hat{\mathbf{q}}_n$.

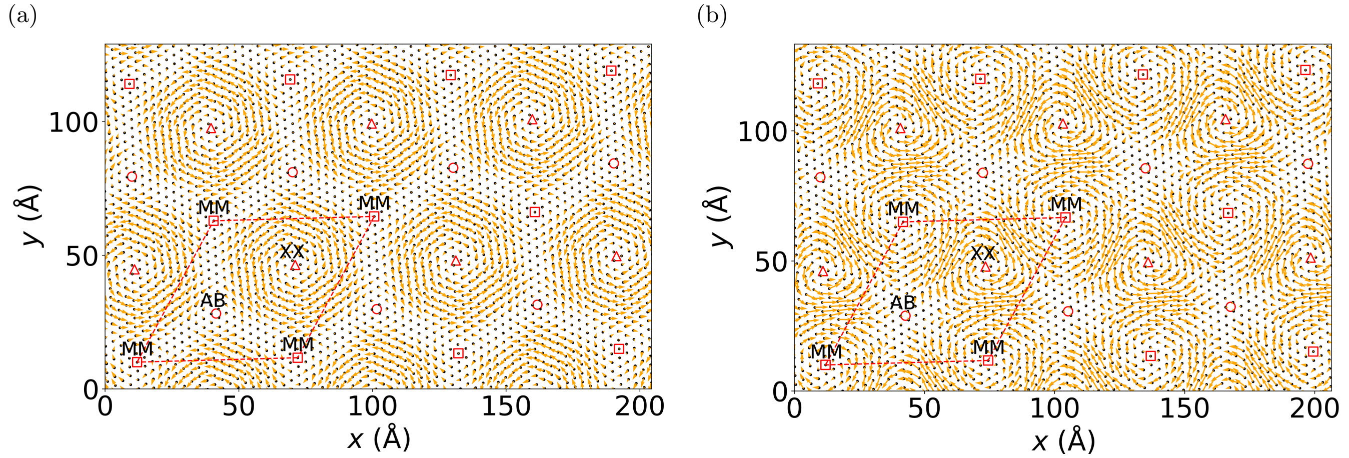

SUPPLEMENTARY FIG. S1: In-plane atomic relaxations of twisted bilayers $MoSe_2$ and $NbSe_2$. In-plane displacements of metal atoms relative to unrelaxed initial structure in the top layer for (c) twisted bilayer $MoSe_2$ and (d) twisted bilayer $NbSe_2$. The red circles denote the centers of the AB stacking regions (metal on top of chalcogen and vice versa), the red squares denote the centers of the MM stacking region (metal on top of metal), and the red triangles denote the centers of the XX stacking region (chalcogen on top of chalcogen).

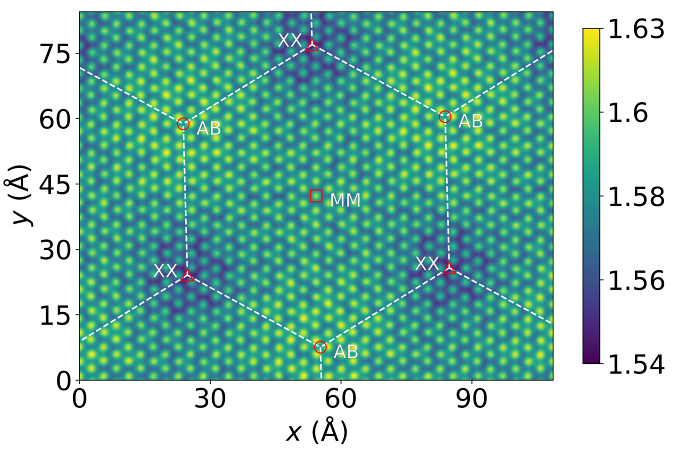

SUPPLEMENTARY FIG. S2: The smeared Mo atomic density in the bottom layer of relaxed twisted $MoSe_2/MoSe_2$. The red cirles, triangles, and squares mark the centers of the XX, AB and MM stacking regions respectively. White dashed lines join the AB and XX centers for visual aid.

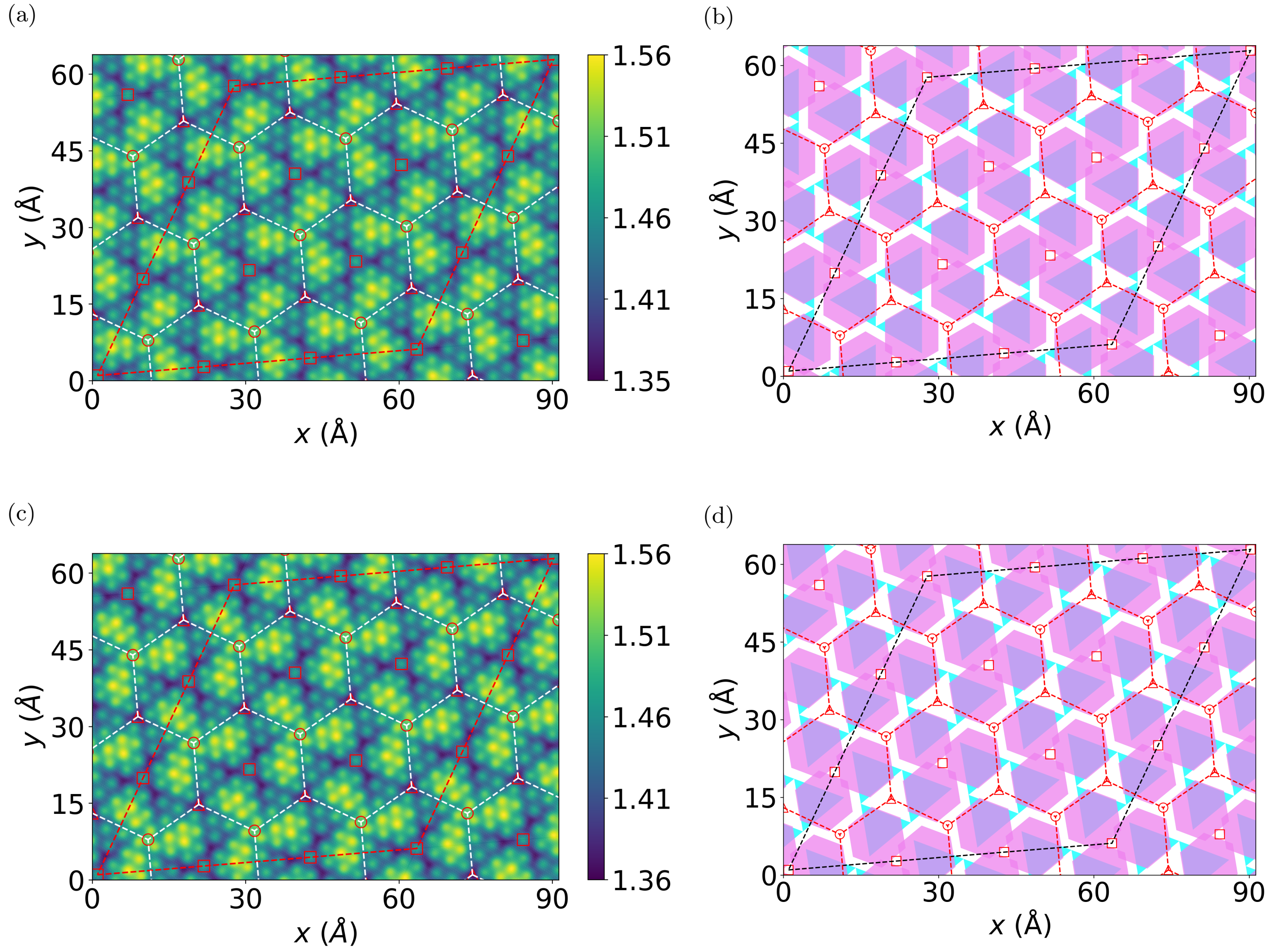

SUPPLEMENTARY FIG. S3: Charge density wave in twisted bilayer $NbSe_2$ at a twist angle of 9.43° from a supercell calculation. Smeared Nb atomic density of (a) the bottom layer and (c) the top layer. The red parallelogram represents the 3×3 supercell of moiré unit cells used in the calculation. The white dashed lines join the AB and XX centers for visual aid. Motifs corresponding to filled-center triangular (hexagonal) CDWs are shown as cyan (purple) symbols for (b) the bottom layer and (d) the top layer. The black parallelogram represents the 3×3 supercell of moiré unit cells used in the calculation. The red circles denote the centers of the AB stacking regions (metal on top of chalcogen and vice versa), the red squares denote the centers of the MM stacking regions (metal on top of metal), and the red triangles denote the centers of the XX stacking regions (chalcogen on top of chalcogen). The red dashed lines join the AB and XX centers for visual aid.

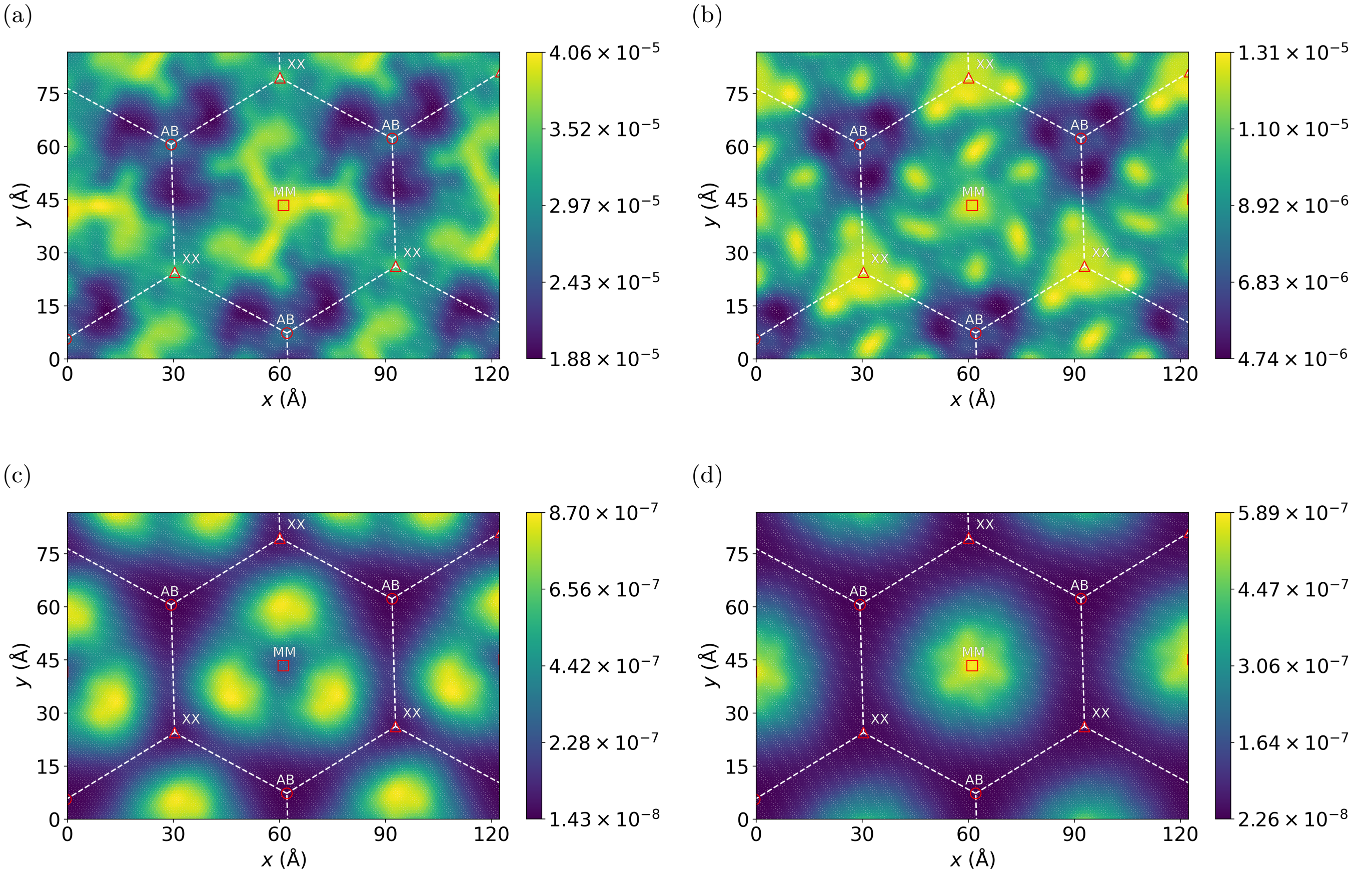

SUPPLEMENTARY FIG. S4: The simulated constant-height STM images for twisted bilayer $NbSe_2$ at the twist angle of $\theta = 3.14^\circ$ taken at a height of 10.3 Å, which is slightly above the topmost Se atom. The STM images were taken at (a) -1.09 eV, (b) -0.37 eV, (c) 1.09 eV, (d) 1.19 eV from the Fermi level.

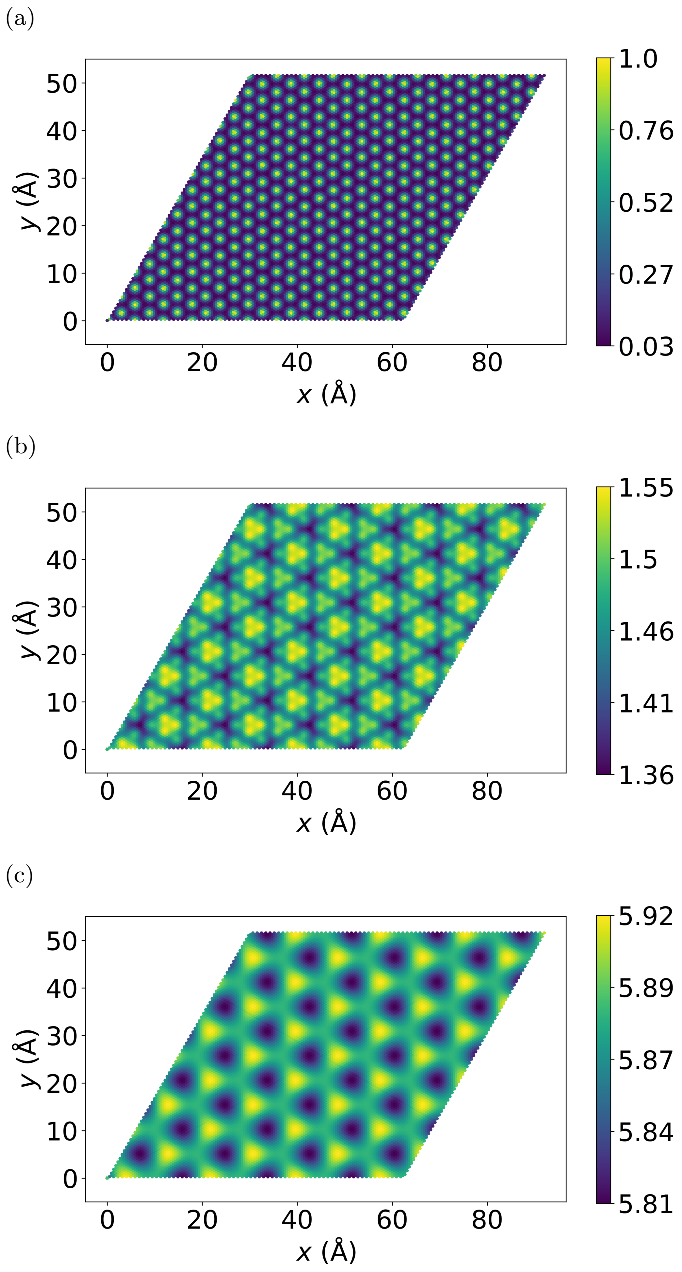

SUPPLEMENTARY FIG. S5: The smeared Nb atomic densities for a monolayer with a 3×3 hollow-center charge density wave of amplitude 0.07 Å for different values of the smearing parameter σ: (a): 0.2 a_0, (b): 0.45 a_0, and (c) 0.9 a_0 with $a_0 = 3.44$Å.

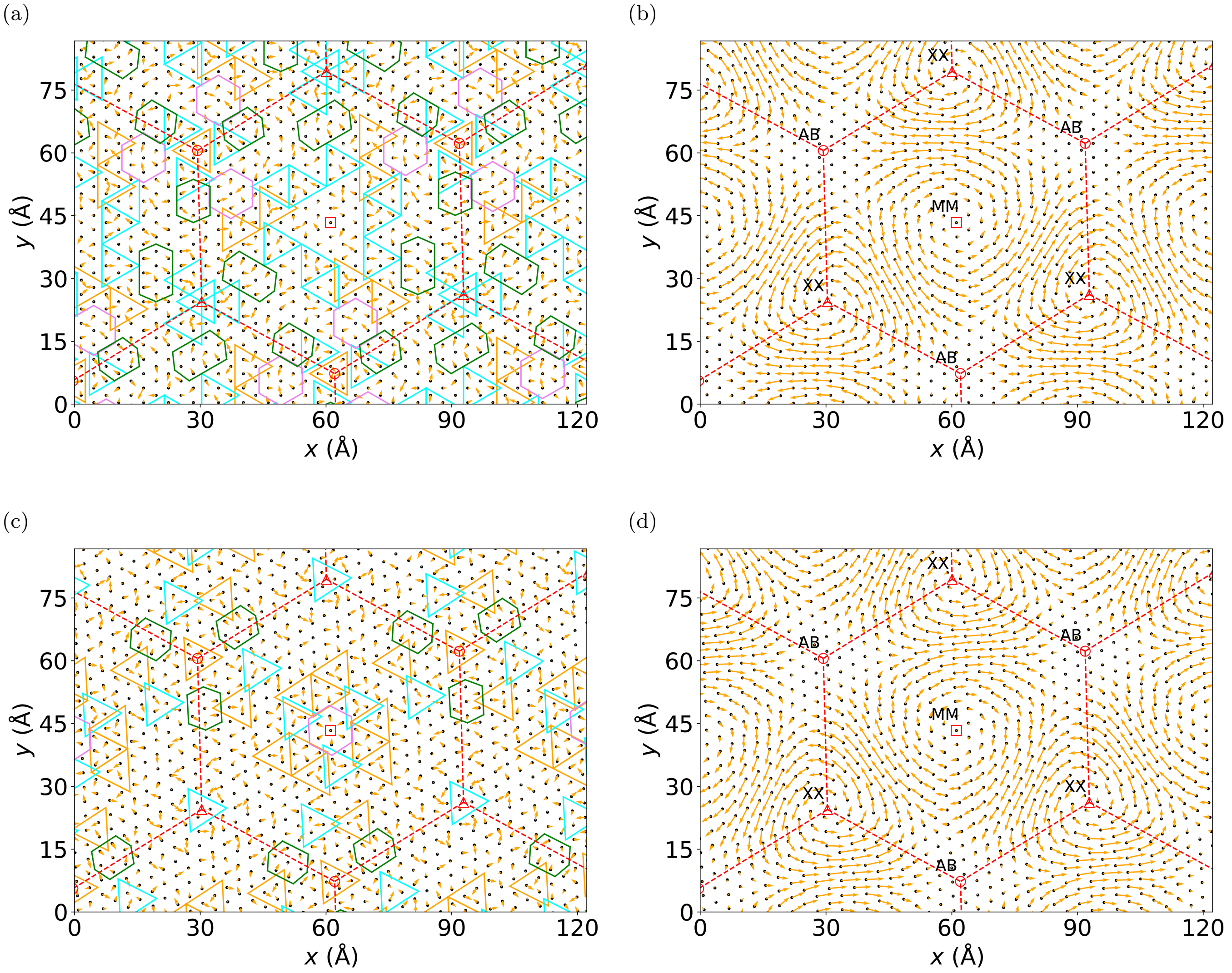

SUPPLEMENTARY FIG. S6: The charge density wave displacements for the bottom layer (a) and the top layer (c). The moiré displacements obtained using for the bottom (b) and the top layer (d). Purple hexagons, cyan triangles, orange trianges, green strips incides hexagonal CDW motifs, filled-center CDW motifs, hollow-center CDW motifs and stripe CDW motifs, respectively. The red circles denote the centers of the AB stacking regions (metal on top of chalcogen and vice versa), the red squares denote the centers of the MM stacking regions (metal on top of metal), and the red triangles denote the centers of the XX stacking regions (chalcogen on top of chalcogen). The red dashed lines join the AB and XX centers for visual aid.

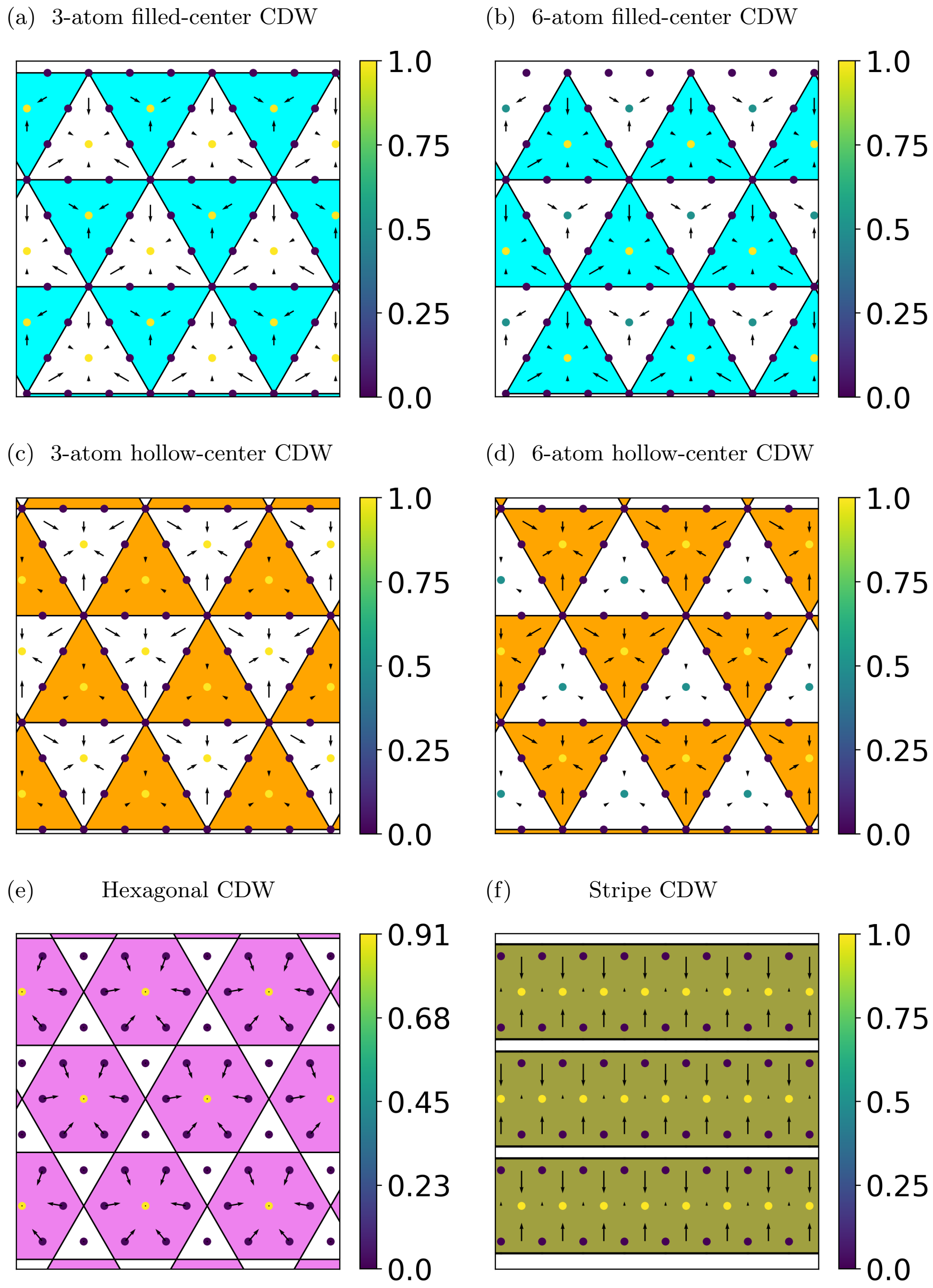

SUPPLEMENTARY FIG. S7: Order parameters for the different charge density wave motifs and corresponding reference displacement patterns. (a): Order parameter for three-atom triangle of the filled-center CDW evaluated in a monolayer with a filled-center CDW. (b): Order parameter for six-atom triangle of the filled-center CDW evaluated in a monolayer with a filled-center CDW; (c): Order parameter for three-atom triangle of the hollow-center CDW evaluated in a monolayer with a hollow-center CDW. (b): Order parameter for six-atom triangle of the hollow-center CDW evaluated in a monolayer with a hollow-center CDW; (e) Order parameter for hexagonal CDW evaluated for a monolayer with a hexagonal CDW; and (e): Order parameter for a stripe CDW evaluated for a monolayer with a stripe CDW. Colored solid circles denote the positions of the motif centers; i.e. Se atoms in (a) and (b), interstitial sites in (c) and (d); Nb atoms in (e) and the mid-points between neighboring Nb atom pair in (f). The color of the solid circles represents the value of the order parameters. The black arrows represent the displacements of the Nb atoms. The corresponding symbols (cyan triangles for filled-center CDW; orange triangles for hollow-center CDW; purple hexagons for hexagonal CDW and green stripe for stripe CDW) are shown in each of the panels.

REFERENCES

[1] J. G. McHugh, V. V. Enaldiev, and V. I. Fal'ko, "Moiré superstructures in marginally twisted $NbSe_2$ Bilayers," Phys. Rev. B, vol. 108, p. 224111, Dec 2023.

[2] R. Bianco, L. Monacelli, M. Calandra, F. Mauri, and I. Errea, "Weak dimensionality dependence and dominant role of ionic fluctuations in the charge-density-wave transition of $NbSe_2$," Phys. Rev. Lett., vol. 125, p. 106101, Sep 2020.

[3] B. Guster, C. Rubio-Verd, R. Robles, J. Zaldvar, P. Dreher, M. Pruneda, J. . Silva-Guilln, D.-J. Choi, J. I. Pascual, M. M. Ugeda, P. Ordejn, and E. Canadell, "Coexistence of elastic modulations in the charge density wave state of 2H-$NbSe_2$," Nano Letters, vol. 19, no. 5, pp. 3027–3032, 2019. PMID: 30998364.

[4] J. M. Campanera, G. Savini, I. Suarez-Martinez, and M. I. Heggie, "Density functional calculations on the intricacies of moiré patterns on graphite," Phys. Rev. B, vol. 75, p. 235449, Jun 2007.

[5] J. M. Soler, E. Artacho, J. D. Gale, A. Garca, J. Junquera, P. Ordejn, and D. Snchez-Portal, "The SIESTA method for ab initio order-N materials simulation," Journal of Physics: Condensed Matter, vol. 14, p. 27452779, Mar 2002.

[6] V. R. Cooper, "Van der waals density functional: An appropriate exchange functional," Phys. Rev. B, vol. 81, p. 161104, Apr 2010.

[7] N. Troullier and J. L. Martins, "Efficient pseudopotentials for plane-wave calculations," Phys. Rev. B, vol. 43, pp. 1993–2006, Jan 1991.
